# Retraction: EZH2-mediated microRNA-375 upregulation promotes progression of breast cancer via the inhibition of FOXO1 and the p53 signaling pathway

**DOI:** 10.3389/fgene.2025.1655052

**Published:** 2025-07-08

**Authors:** Frontiers Editorial Office

The journal retracts the 29th March 2021 article cited above.

Following publication, concerns were raised regarding the integrity of the images in the published figures. The authors failed to provide a satisfactory explanation during the investigation, which was conducted in accordance with Frontiers’ policies. This retraction was approved by the Chief Editors of Genetics and the Chief Executive Editor of Frontiers. The authors have not responded to correspondence regarding this retraction.

